# Functional Characterization of Hexacorallia Phagocytic Cells

**DOI:** 10.3389/fimmu.2021.662803

**Published:** 2021-07-26

**Authors:** Grace A. Snyder, Shir Eliachar, Michael T. Connelly, Shani Talice, Uzi Hadad, Orly Gershoni-Yahalom, William E. Browne, Caroline V. Palmer, Benyamin Rosental, Nikki Traylor-Knowles

**Affiliations:** ^1^ Department of Marine Biology and Ecology, Rosenstiel School of Marine and Atmospheric Science, University of Miami, Miami, FL, United States; ^2^ The Shraga Segal Department of Microbiology, Immunology, and Genetics, Faculty of Health Sciences, Regenerative Medicine and Stem Cell Research Center, Ben Gurion University of the Negev, Beer Sheva, Israel; ^3^ Ilse Katz Institute for Nanoscale Science and Technology, Ben-Gurion University of the Negev, Beer Sheva, Israel; ^4^ Department of Biology, University of Miami, Coral Gables, FL, United States; ^5^ School of Biological and Marine Sciences, University of Plymouth, Plymouth, United Kingdom

**Keywords:** Hexacorallia, coral immunity, phagocytosis, FACS, innate immunity, coral reefs, flow cytometry, Sea anemone

## Abstract

Phagocytosis is the cellular defense mechanism used to eliminate antigens derived from dysregulated or damaged cells, and microbial pathogens. Phagocytosis is therefore a pillar of innate immunity, whereby foreign particles are engulfed and degraded in lysolitic vesicles. In hexacorallians, phagocytic mechanisms are poorly understood, though putative anthozoan phagocytic cells (amoebocytes) have been identified histologically. We identify and characterize phagocytes from the coral *Pocillopora damicornis* and the sea anemone *Nematostella vectensis*. Using fluorescence-activated cell sorting and microscopy, we show that distinct populations of phagocytic cells engulf bacteria, fungal antigens, and beads. In addition to pathogenic antigens, we show that phagocytic cells engulf self, damaged cells. We show that target antigens localize to low pH phagolysosomes, and that degradation is occurring within them. Inhibiting actin filament rearrangement interferes with efficient particle phagocytosis but does not affect small molecule pinocytosis. We also demonstrate that cellular markers for lysolitic vesicles and reactive oxygen species (ROS) correlate with hexacorallian phagocytes. These results establish a foundation for improving our understanding of hexacorallian immune cell biology.

## Introduction

Innate immunity is an important protective defense response used to recognize and destroy non-self. Through an intricate cellular recognition system, non-self can be recognized and initiate downstream signaling pathways that ultimately lead to effector responses such as phagocytosis, coagulation, and antimicrobial defense (1). Among the Cnidaria, significant progress has been made within Class: Hexacorallia (i.e., scleractinian corals and sea anemones) on our understanding of innate immune system responses to environmental stress (2–8). Coral reefs are currently one of the most endangered ecosystems on the planet due to anthropogenic climate change and local human impacts (9–14). These anthropogenic impacts have caused an increase in disease outbreaks and virulence, as well as an increase in bleaching events, all culminating in negative impacts on coral immune function (10, 11, 13–18).

Within Hexacorallia, our primary understanding of the immune system comes from previous studies examining histology, enzymatic reactions, and the genomic response to synthetic immune stimulators, disease pathogens, heat stress, and wound healing (1, 19–38). For example, in stony corals, constituent immunity as measured by melanin reactivity, fluorescent protein expression and prophenoloxidase activity, has an inverse relationship to bleaching mortality and disease susceptibility, indicating that the immune system plays a critical role in the outcome of these reactions (19). Furthermore, we know that many candidate immune genes including those involved in pathogen recognition, proteolytic response, coagulation, antimicrobial peptide precursors, and the regulation of inflammation and apoptosis are expressed in response to both coral bleaching events and disease exposure, supporting the hypothesis that innate immune responses are associated with these environmental stressors (5, 6, 8, 25, 31, 39).

At a cellular level, anthozoan innate immunity has been well documented using histological methods (1, 6, 19–23, 30, 32–38, 40). In response to wound healing, bleaching, and disease, putative phagocytes termed amoebocytes migrate to lesion sites (34, 40–43). Additionally, in response to microplastic exposure, gastrodermal cells that exhibit amoebocyte behavior uptake microplastics (44–49). Phenotypically, these cells show hallmarks of immune cell morphology such as high intracellular granularity in combination with ameboid morphology (34, 40–43). Lastly, phagocytosis has been identified as the primary mechanism required to initiate the establishment of intracellular symbiosis between hexacorallian cells and the Symbiodiniaceae dinoflagellates (50–59). While abundant evidence points to phagocytosis as a critical cellular response in Hexacorallia, many of the functional cellular mechanisms of phagocytosis in hexacorallians are still not well understood.

Phagocytosis is the primary mechanism by which specialized immune cells, termed “professional phagocytes” ingest and destroy target particles such as foreign pathogens and damaged cells (60, 61). In mammalian systems, five specialized types of professional phagocytes have been well described. In many invertebrate systems, including Hexacorallia, distinct immune cell types and the accompanying phagocytic functional mechanisms have not been as well described. Novel cell structures involved in phagocytosis have recently been discovered within *Nematostella vectensis* (62, 63) and only recently have phagocytic cells been described in Ctenophora (64).

During the process of phagocytosis, target particles are recognized by receptors on immune cells. Upon recognition, target particles are endocytosed into specialized intracellular vesicles, called phagosomes, for degradation. This process requires a rapid rearrangement of the actin cytoskeleton to produce pseudopodial extensions, which participate in target particle engulfment (65). Additional lysosomal vesicles then fuse with the phagosome, resulting in a reduction of intravesical pH and the release of digestive enzymes. The end products of phagosome-mediated degradation are then exocytosed (66, 67). This vesicular activity is typically accompanied by the production of reactive oxygen species (ROS), generating a “respiratory burst” through the activation of the NADPH oxidase complex within the phagocyte, further aiding in the degradation of engulfed material (61).

In this study we characterize several key mechanistic properties of phagocytosis in two hexacorallians, the coral *Pocillopora damicornis*, and the estuarine sea anemone, *Nematostella vectensis* (68, 69). Using fluorescence-activated cell sorting (FACS) and microscopy, we show that a distinct population of phagocytic cells are competent to engulf bacteria and carboxylated fluorescent beads. We further show that cytoskeletal inhibitors interfere with efficient particle phagocytosis but do not affect small molecule pinocytosis. We also demonstrate that cellular markers for lysolitic vesicles and ROS activity are present in hexacorallian phagocytes. This study addresses a critical gap in our understanding of hexacorallian cellular immune system activity during phagocytosis and establishes new tools for assessing immune system function in hexacorallians. Understanding the cellular mechanisms of phagocytosis is one of the first steps of thoroughly characterizing the metazoan immune system.

## Methods

### Animal Husbandry

The *P. damicornis* clonal fragments used in this study originated from a single Panamanian genotype that has been housed at the Rosenstiel School of Marine and Atmospheric Science since early 2005 (68). Fragments were kept in indoor flow-through tanks with 12-hour light dark cycles. *N. vectensis* individuals were generously provided by Prof. Tamar Lotan from Haifa University and Prof. Yehu Moran from the Hebrew University and kept in dishes of 11 ppt artificial seawater (ASW) at an 18°C incubator at the mariculture room at the Regenerative Medicine and Stem Cell Research Center, Ben Gurion University (Approved by the Israel ministry of agriculture and university biosafety committee) (70).

### Cell Dissociation

*P. damicornis* tissue was dissociated from healthy 1 cm branches using an airbrush (Paasche H Series) with a FACS staining media consisting of calcium and magnesium free 3.3 X phosphate buffer saline (PBS), 2% heat-inactivated fetal bovine serum (FBS), and 20 mM HEPES buffer into a sterile collection bag. Cells were then filtered through 70 µm and 40 µm cell strainers, and thereafter kept on ice (71, 72).

To dissociate *N. vectensis*, individual animals were mechanically dissociated using a sterile razor blade and filtered through 100 µm and 40 µm cell strainers. A syringe plunger was used to help facilitate the filtering. The staining media used was L-15 based and consisted of 2% heat inactivated fetal bovine serum (FBS), 20 mM HEPES. It was then brought to 1.42 X PBS molarity using calcium and magnesium free 10 X PBS and supplemented with 0.05% of NaN_3_ to reduce contamination. The entire cell dissociation process was done on ice to lower cell metabolism and minimize cell damage. Cells were then washed by centrifugation at 500 x g at 4°C for 5 min.

### Assay Preparation and Flow Cytometry

Cells were brought to an approximate concentration of 1 - 4 x 10^6^ cells/mL in FACS staining media. The cell concentration was estimated by using either a 0.05% trypan blue staining and counting on a hemocytometer or using a flow cytometer to count cells in a set volume. The trypan blue concentration was determined as the maximum concentration that can be used without causing cell aggregation due to the high salinity. After determining the concentration, cells were used in either phagocytosis or pinocytosis assays.

#### Phagocytosis and Pinocytosis Assays

For both *P. damicornis* and *N. vectensis*, phagocytosis assays were done in 96 well U-shaped plates, with 100,000 cells/well in 200 µl of FACS media. Phagocytic assays consisted of exposure to several different assays. These included: carboxylated beads, bacteria, or a fungal antigen. Beads of different sizes and fluorescent colors were used at a ratio to cells of 4:1 (Fluoresbrite YG Carboxylate Microspheres (yellow-green and carboxylated), 1 µm and 4 µm, and Polychromatic (PC Red) Red Microspheres, 1 µm; Polysciences). For the quenching of un-engulfed beads, trypan blue assay was used at 1mg/ml for 30 min before FACS analysis. For the bacterial challenges, inactive particles of *Staphylococcus aureus* (pHrodo™ Green *S. aureus* Bioparticles™ Conjugate for Phagocytosis; Thermo Fisher Scientific) and *Escherichia coli* (pHrodo™ Red E. coli BioParticles™ Conjugate for Phagocytosis; Thermo Fisher Scientific), were tested at 15 µg/ml. Zymosan, (pHrodo™ Red Zymosan Bioparticles™; Thermo Fisher Scientific) a fungal antigen, was tested at 15 µg/ml. For both the bacteria and zymosan, the fluorescence is bright only upon vesicle fusion with low pH vesicles, indicating the creation of phagolysosomes. Lastly, 15 ug/ml of DQ™ ovalbumin was used as a fluorogenic substrate for proteases (Thermo Fisher Scientific). The ovalbumin is labeled with BODIPY dyes which quench each other. Upon hydrolysis of the DQ™ ovalbumin to single dye-labeled peptides by proteases, quenching is relieved, producing green fluorescence. This occurrence is indicative of active degradation within the cell and can be observed by FACS and microscopy.

Next, we tested in both *P. damicornis* and *N. vectensis*, whether hexacorallian phagocytic cells could engulf damaged cells that derived from themselves. The *N. vectensis* and *P. damicornis* control groups were stained with CellTrace, and the *N. vectensis* and *P. damicornis* experimental group were stained with CFSE and lipophilic stains DiO and DiI. The experimental group was divided into ambient conditions for 1 hr and 42oC heat stress conditions for 1 hr. After the heat stress or ambient incubation for the experimental group was complete, the experimental (heat stressed or ambient) and control groups were combined and incubated for 3 hours.

Pinocytosis was stimulated using large dextran molecules, a complex sugar molecule derived from bacteria (0.65 µg/ml; Fluorescein Isothiocyanate - Dextran; molecular weights of 40,000, 120,000, 500,000, 2,000,000 MW; Sigma-Aldrich). *P. damicornis* cells were incubated in the assays for 3 hours and *N. vectensis* cells were incubated overnight.

To inhibit pseudopodia, the actin filament blockers cytochalasin B, cytochalasin D, and latrunculin A were applied to the cells immediately prior to challenge assay exposure (60 µM, Alfa Aesar; 60 µM, Sigma-Aldrich; 7 µM, Abcam, respectively). In *P. damicornis*, only latrunculin A was found to be effective for blocking actin filament formation and was therefore used exclusively. Actin filament formation was blocked prior to challenge assays for both phagocytosis and pinocytosis assays.

After phagocytosis/pinocytosis incubation, cells were labeled with 0.2 µM of LysoTracker, a marker for lysolitic vesicles (Thermo Fisher Scientific) and incubated for 30 minutes prior to flow cytometry analysis. *N. vectensis* cells were pre-labeled with CellTrace Far Red in a serum-free media (1 µM, Thermo Fisher Scientific) for 1 hour at 18°C before phagocytosis assays with or without actin inhibitor exposure.

#### Detection of Lysolitic- and ROS-Containing Vesicles in Cells

A separate experiment was conducted to test whether cells with a higher concentration of lysolitic vesicles and increased ROS production were associated with increased phagocytic activity. Following cell concentration estimation, cells were incubated for 45 minutes in either 0.2 µM LysoTracker (Thermo Fisher Scientific) or 5 µM of CellROX (Thermo Fisher Scientific), which are markers for low pH and ROS, respectively. After incubation, cells were centrifuged for 5 minutes at 250 x g and resuspended in 500 µL of FACS staining media and analyzed on a Sony SH800 (Sony MA900 for *N. vectensis*) flow cytometer fitted with a 100 µm nozzle. Gate selection for cell sorting included cell populations with low and high staining signals for LysoTracker and CellROX (the highest and lowest 25%). Gated cells were collected into 2 mL of FACS staining media. Sorted cells were then exposed at a 4:1 ratio of cells to Fluoresbrite YG Carboxylate Microspheres and incubated for 16 hours (72). Following incubation, cells were then labeled with LysoTracker for 30 minutes at 4°C prior to further flow cytometry analyses. Cellular debris and background noise were removed from the analyses, using unstained cell slurries and pure bead samples of 1 µm as size references. All FACS analyses were conducted using FlowJo v10 (BD Becton, Dickinson).

#### Imaging Flow Cytometry

Cells were analyzed using the ImageStream X Mark II Imaging flow cytometer (Amnis, Co., Seattle, WA, USA) with a 40x/0.75 objective. Data from channels representing bright field, fluorescence (green COMPLETE FLUOROPHORE excitation at 488 nm), and fluorescence (red COMPLETE FLUOROPHORE excitation at 642 nm) were recorded for 10,000 cells for each analyzed sample. IDEAS^®^ software was then used to generate the quantitative measurements of focused, single cells, and fluorescent signal quantification.

### Microscopy

Cells were sorted into 500 µL of FACS staining media, centrifuged for 5 minutes at 200 x g and resuspended in 10-50 µL of FACS staining media. Then 2ul of resuspended cells were mounted onto a micro-welled slide with a coverslip for imaging. For *P. damicornis*, images were acquired using a ZEISS Axio Imager.Z2 with a ZEISS AxioCam MRm Rev3 camera and analyzed using ZEISS Zen Blue software. For *N. vectensis*, images were acquired using a ZEISS LSM900 confocal microscope and analyzed using ZEISS ZEN-black software.

## Results

### Identification of Multiple Hexacorallian Phagocytic Cell Morphologies

**Figure 1:**
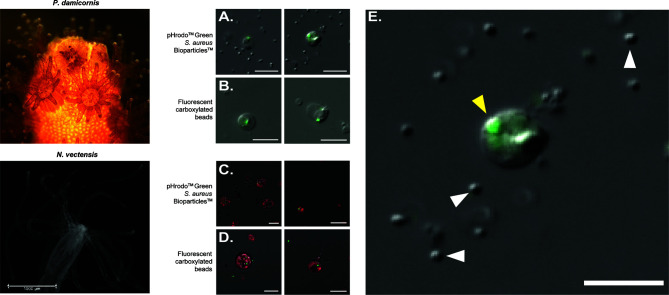
FACS-isolated cells of *Pocillopora damicornis* and *Nematostella vectensis* positive for phagocytosis. **(A–E)** Images of sorted cells positive for *S. aureus* and bead assays. **(A)** Images of sorted cells from *P. damicornis* challenged with inactive pHrodo™ *S. aureus* particles, with the center host cells having internalized, pH-activated bacteria. **(B)** Images of sorted cells from *P. damicornis* challenged with fluorescent carboxylated beads, with the center host cells having internalized beads. **(C)** Images of sorted cells from *N. vectensis* challenged with inactive pHrodo™ *S. aureus* particles, with the CellTrace-stained animal cells having internalized, pH-activated bacteria. **(D)** Images of sorted cells from *N. vectensis* challenged with fluorescent carboxylated beads, with the CellTrace-stained cells having internalized beads. **(E)** Enlarged version of right image in **(A)**, a *P. damicornis* cell challenged with *S. aureus*. Yellow arrow shows an internalized, pH-activated *S. aureus* particle that fluoresces bright green at a low pH. White arrows indicate the cocci bacterial particles. *S. aureus* particles are fluorescent after the fusion of the bacteria with the lysolitic vesicle, which leads to lowered pH. This is not seen in the free bacteria that are not internalized by the cells and can be seen only with DIC. All *P. damicornis* pictures were taken on a ZEISS Axio Imager.Z2 microscope with a magnification of 40X using a combination of DIC and 470nm LED with ZEISS 38HE filter set. All *N. vectensis* pictures were taken on ZEISS LSM900 confocal microscope with a magnification of 40X using a combination of PMT, green and far-red filters. White scale bars represent 10 μm.

In order to isolate phagocytic cells from *P. damicornis* and *N. vectensis*, we performed two phagocytic assays, incubating cell suspensions with either carboxylated fluorescent beads or inactive *S. aureus* particles. Cells positive for fluorescent labeling were then sorted and observed by fluorescent microscopy (**Figures 1**, **S1**). In both species, the sorted cell populations positive for either microplastic beads or *S. aureus* fluorescence exhibited two distinct cell morphologies, resembling either a granular spheroid or ameboid phenotype (**Figure 1**). Filopodial extensions were also occasionally observed (**Video S1**). *P. damicornis* cells averaged approximately 10-20 µm in diameter while *N. vectensis* positive cells averaged 10-15 µm in diameter. Additional 3D image analysis and imaging flow cytometry on *N. vectensis* cells with engulfed bacteria and beads provide further evidence that the target particles were engulfed and not exterior to the cell surface (**Figures S2** and **S3**). Importantly, only engulfed *S. aureus* particles show bright green fluorescence (compared to the un-engulfed bacteria that remain outside of the cells), suggesting the engulfment of the bacteria is combined with fusion to lysoltic vesicles, creating a low pH environment within the phagolysosome, which is required to activate the conjugated pHrodo fluorophore (**Figures 1A, C, enlarged in E**, **S2**).

### Phagocytosis of Diverse Antigens, Their Fusion With Low pH Vesicles, and Protease Degradation Activity

**Figure 2:**
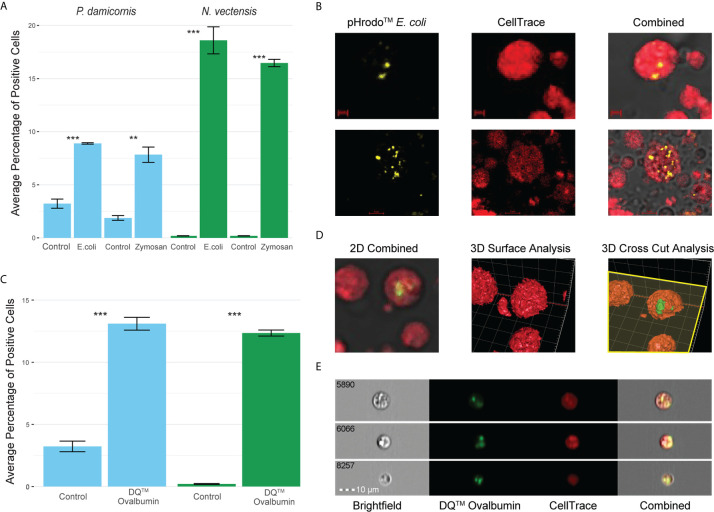
Phagocytosis in *P. damicornis* and *N. vectensis* of different antigens, and their fusion with low pH vesicles and protein degradation. **(A)** Percentage of cells in *P. damicornis* (blue bars) and *N. vectensis* (green bars) that engulfed pHrodo™ *E. coli* (left) and pHrodo™ zymosan (right) particles. Each test treatment is significantly different from the respective control treatment. **(B)** Images of isolated *N. vectensis* cells challenged with pHrodo-red™ *E. coli* particles, taken by confocal microscopy. *Left:* internalized, pH-activated *E. coli* particles that fluoresce brightly in acidic environments. *Middle: N. vectensis* stained with CellTrace. *Right:* Overlays of left and middle panels in confocal microscopy PMT. *E. coli* particles fluoresce after the fusion with low pH lysolitic vesicles. Scale bar for upper panels 2 μm, and 5 μm for lower panels. **(C)** Percentage of cells in *P. damicornis* (blue bars) and *N. vectensis* (green bars) that engulfed DQ™ ovalbumin, a particle that only fluoresces bright green when hydrolysis by proteases due to the quenching of the reagent. The number of cells engulfing the ovalbumin is significantly higher than that of the control treatment. **(D)** 3D analysis of a *N. vectensis* cell stained with CellTrace and has been exposed to DQ™ ovalbumin. *Left:* 2D overlay of confocal microscopy and PMT, bar-2 μm. *Middle:* 3D surface analysis of the same *N. vectensis* cell featured in the left panel and shows the absence of DQ™ ovalbumin from the surface of the cell. *Right:* 3D cross-sectional analysis of the same *N. vectensis* cell featured in the left and middle panels and shows fluorescing DQ™ ovalbumin particles within the cell. The yellow plane depicts the cross section done in the Z-axis (right panel), and surface analysis of the parts below the plane to show what is inside the cells. Grid scales are 3 μm. **(E)** Inserts of ImageStream analysis of *N. vectensis* cell stained with CellTrace (red) and has been exposed to DQ™ ovalbumin (green upon protein hydrolysis), scale bar 10 μm. P-value marks: **p < 0.01, ***p < 0.001.

In order to see if phagocytic cells from *P. damicornis* and *N. vectensis* would engulf different types of pathogen-associated antigens, we tested pHrodo™ *S. aureus*, pHrodo™ *E. coli* and pHrodo™ zymosan. All were significantly engulfed by a subpopulation of cells (**Figure 2A**), with comparable numbers to those engulfing beads. Both *E. coli* and zymosan were labeled with pHrodo for the validation of particle intake and fusion with low pH vesicles, as seen in the confocal imaging (**Figure 2B**).

For validation of phagocytic target degradation, DQ™ Ovalbumin was used (**Figures 2C–E**). We saw significant engulfment and degradation of the ovalbumin (**Figure 2C**) and validated its intake by confocal 2D and 3D surface analysis (**Figure 2D**). Moreover, imaging flow cytometry analysis showed that the degradation of phagocytosis targets happens in compartments in the phagocytic cells, suggesting the creation of phagolysosomes (**Figure 2E**).

### Hexacorallian Phagocytes Engulf Damaged Cells

**Figure 3:**
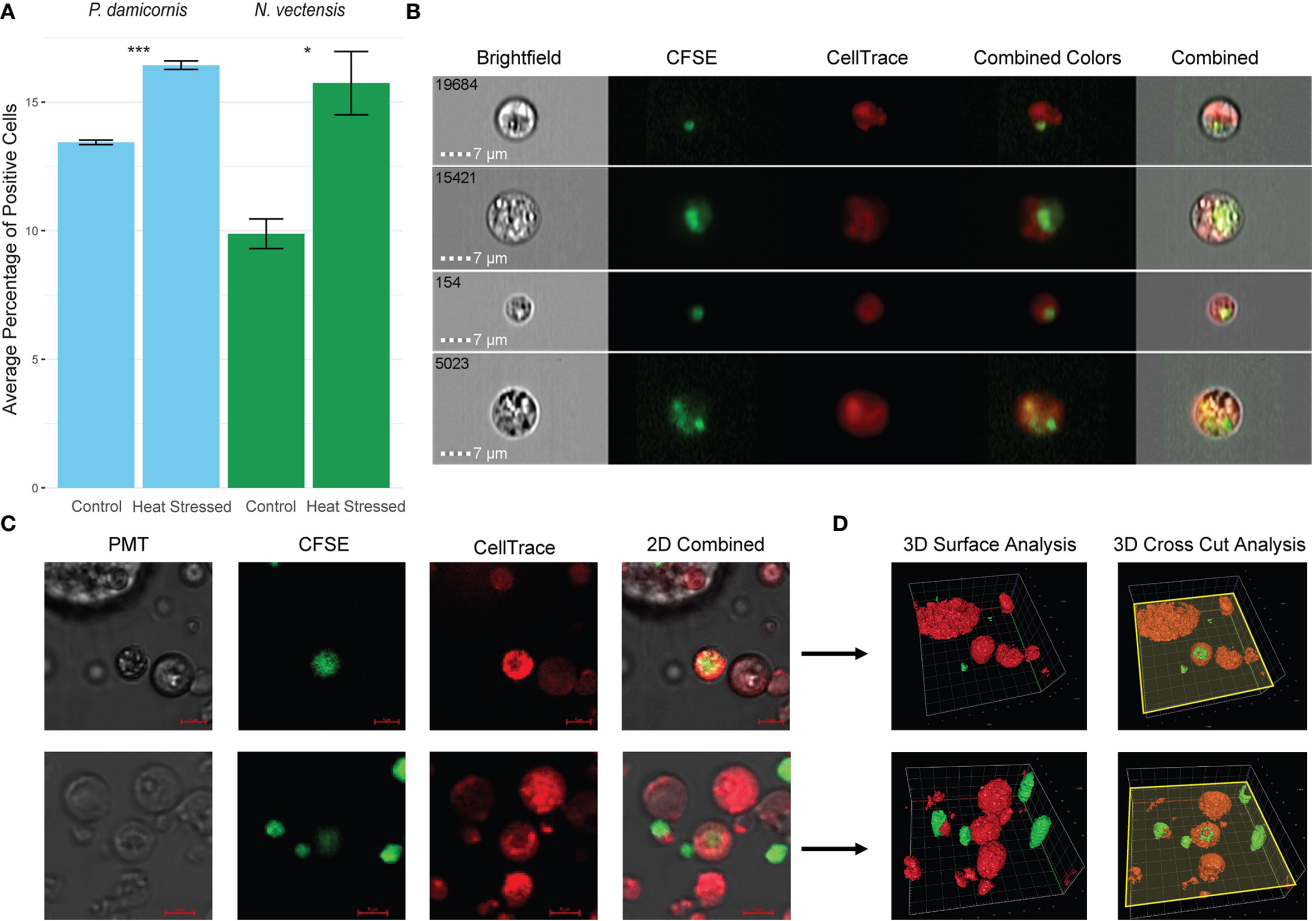
Phagocytosis in *P. damicornis* and *N. vectensis* of damaged cells. **(A)** Percentage of cells in *P. damicornis* (blue bars) and *N. vectensis* (green bars) that engulfed cells. Cells from different animals were differentially labeled (CellTrace and CFSE), to detect double positive events that would indicate engulfment of cells. The control group was exposed to cells kept at ambient conditions, while the heat stress group (both labeled with CFSE) was exposed to cells that were kept at 42°C for one hour. Each test treatment is significantly different from the control treatment. **(B)** Inserts of ImageStream analysis of *N. vectensis* cells stained with CellTrace (red) and cells stained with CFSE (green) that were heat stressed. The images are examples of the gated double positive population for validation of cells, or their particles engulfed by the red cells. Scale bar 7 μm. **(C)** Example of 2D images slides in confocal microscopy of *N. vectensis* cells stained with CellTrace (red) and cells stained with CFSE (green) that were heat stressed. For validation at least 40 slices were taken on a Z axis to create 3D surface analysis (in **D**). Scale bars 5 μm. **(D)** Inserts of 3D surface analysis to show the full cells (right panels), of the examples in **(C)** The yellow plane depicts the cross section done in the Z-axis (right panels), and surface analysis of the parts below the plane to show what is inside the cells. In the center of the image CFSE labeled cells can be seen engulfed by CellTrace labeled cells. Grid scales for the upper panel are 4 μm, and 3 μm for the lower panel. P-value marks: *p < 0.05, ***p < 0.001.

To test whether hexacorallian phagocytic cells engulfed damaged cells derived from themselves, we used heat stress to induce cellular damage. Engulfment of the heat stressed experimental group by the control group cells was observed and analyzed (**Figure 3**). A significant increase in the engulfment of heat stress experimental cells was observed, suggesting that damaged cells are being engulfed by the phagocytes (**Figure 3A**). Further validation of this observation was performed in *N. vectensis* with imaging stream analysis (**Figure 2B**), as well as confocal 2D and 3D surface analysis with Z-axis cross sectioning where engulfment and total internalization of the damaged cells is observed (**Figures 3C, D**).

### Cells Show Functional Characteristics of Phagocytosis

**Figure 4:**
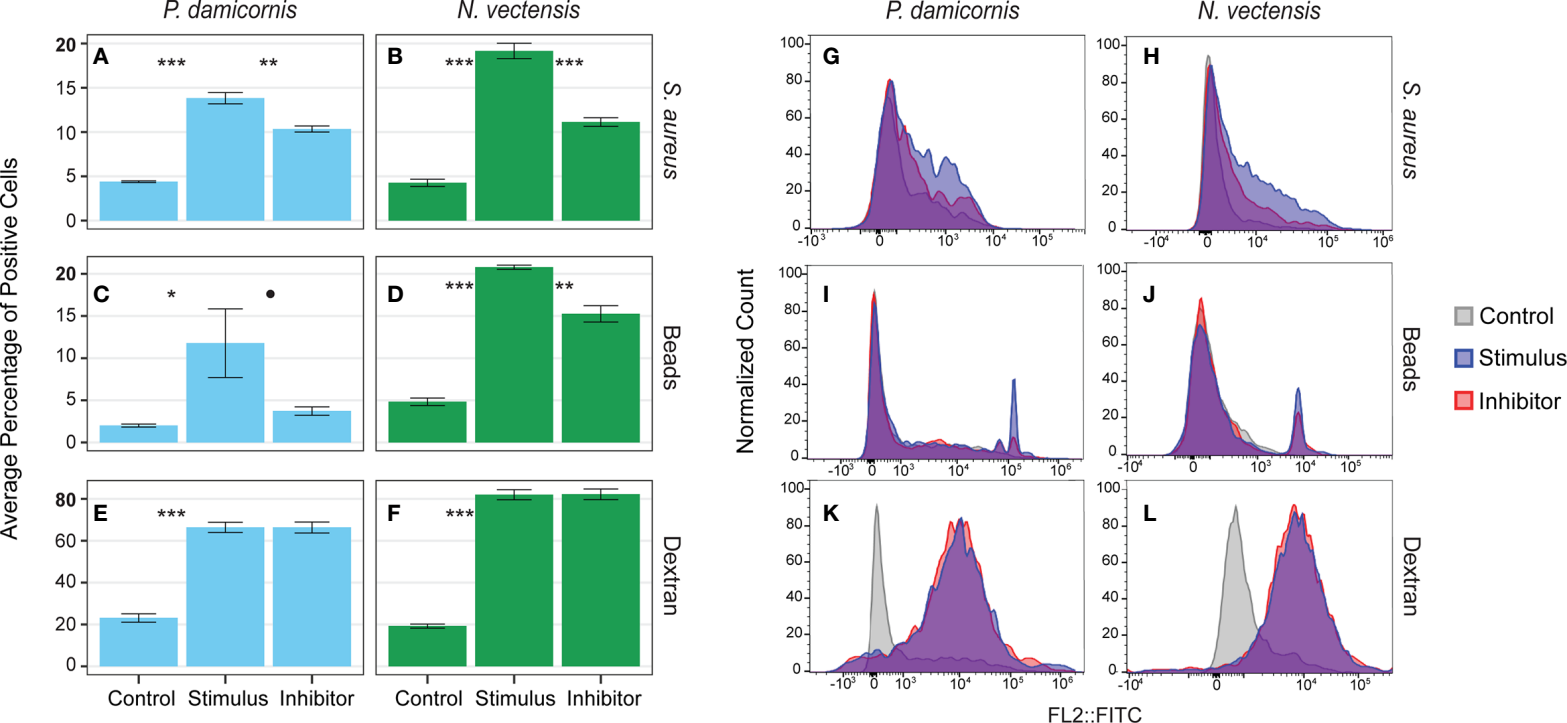
Phagocytosis is a distinct process from pinocytosis and is reliant on actin filament movement. **(A, C, E)** show the percentage of *P. damicornis* cells positive for each assay while **(B, D, F)** show *N. vectensis*. Error bars are representative of the standard error of the mean. **(A, B)** Cellular uptake of inactive *S. aureus* particles is significantly higher in comparison with unexposed controls (p-value in both species < 0.001). The cytochalasin inhibitor resulted in a statistically significant reduction of cellular uptake of inactive *S. aureus* particles (*P. damicornis:* 25% reduction, p-value < 0.01; *N. vectensis*: 42% reduction, p-value < 0.001). **(C, D)** Similar to the inactive *S. aureus* particles assay, cellular uptake of green carboxylated beads is significantly higher in the stimulus group compared to an unexposed control (*P. damicornis* p-value < 0.05; *N. vectensis* p-value < 0.001). The cytochalasin inhibitor resulted in a reduction of cellular uptake of green carboxylated beads, which was significant in *N. vectensis* (27% reduction, p-value < 0.01), but only at 90% confidence in *P. damicornis* (68% reduction, p-value = 0.072). **(E, F)** 66.5% of cells in *P. damicornis* and 82% of cells in *N. vectensis* were detectable after treatment of dextran particles, indicating that particle uptake was ubiquitous. The cytochalasin inhibitor did not cause a statistically significant decrease in dextran uptake, indicating that this process is not facilitated by phagocytosis, but rather pinocytosis. **(G–L)** FACS data for each assay on a histogram of the single filter (FITC). **(G–J)** In the phagocytic assays, the inhibitor sample shows a similar histogram profile as the stimulus, with a reduction in cells with high levels of fluorescence. **(K, L)** The pinocytic assay of dextran exposure shows nearly no difference between the stimulus and inhibitor samples. P-value marks: *p < 0.05, **p < 0.01, ***p < 0.001.

Phagocytosis events were identified by the engulfment of carboxylated beads and inactive *S. aureus* Bioparticles. To test whether engulfment was by active phagocytosis or pinocytosis, the percentage of cells engulfing particles was compared to the percentage of cells that took up fluorescently labeled sugar molecules (500,000 MW dextran-FITC; **Figures 4A–F**). In *P. damicornis*, the percentage of total live cells that phagocytosed target beads or bacteria was 12% - 14%, while 66% of total cells were found to pinocytose dextran (**Figures 4A–F**). Similarly, in *N. vectensis*, the percentage of total live cells that phagocytosed target beads or bacteria was 21% -19% while 82% of total cells were found to pinocytose dextran. In both species, percentages of live cells competent for phagocytosis of target particles was significantly lower than the percentage of cells competent for small molecule pinocytosis (**Figures 4A–F**; all p-values = <0.001). For supplemental validation, an additional bead size was also tested (4 µm diameter), which showed phagocytic reduction for both species. In contrast, the variable dextran molecule sizes (40,000, 150,000 and 2,000,000 MW) showed little difference in pinocytosis (**Figures S4A, B**). Moreover, the use of red fluorescent beads in combination with dextran showed no correlation between dextran and bead intake (**Figure 4C**). These results support the presence of specialized cells responsible for phagocytosis, as compared to the general ability of most cells to uptake large sugar molecules *via* pinocytosis.

### Inhibition of Dynamic Cytoskeletal Rearrangements Affects Phagocytosis but Not Pinocytosis

Actin filament inhibition significantly reduced the phagocytosis of beads and bacteria in *N. vectensis*, and significantly reduced the bacteria phagocytosis in *P. damicornis*. For bead phagocytosis, inhibition in *P. damicornis*= above 90% confidence was reached (*P. damicornis* p-value = 0.07 and 0.01; *N. vectensis* p-value = 0.01 and 0.01, respectively). Actin filament inhibition did not, however, reduce the uptake of sugars *via* pinocytosis (*P. damicornis* p-value > 0.999; *N. vectensis* p-value = 0.960) (**Figures 4G–L**). In *P. damicornis*, the levels of total cells with engulfed beads and bacteria dropped significantly when exposed to latrunculin A, while the percentage of cells that took up dextran did not change (**Figures 4G–L**). Compared to *S. aureus* which fluoresce only within low pH vesicles, beads can fluoresce inside and outside the cell. A trypan blue quenching assay was used to test for un-engulfed beads; some reduction was observed for both species but still significantly higher than the controls (**Figure 4D**). In *N. vectensis*, a combination of cytochalasin B, cytochalasin D, and latrunculin A caused a significant reduction in bead and bacteria engulfment, but no change in dextran uptake. These results suggest that phagocytic activity in these two hexacorallian species requires actin-based cytoskeletal rearrangements to produce the pseudopodial extensions associated with large particle engulfment in contrast to small molecule pinocytosis.

### Lysolitic Vesicles and High Levels of ROS Are Present in Hexacorallian Phagocytes

**Figure 5:**
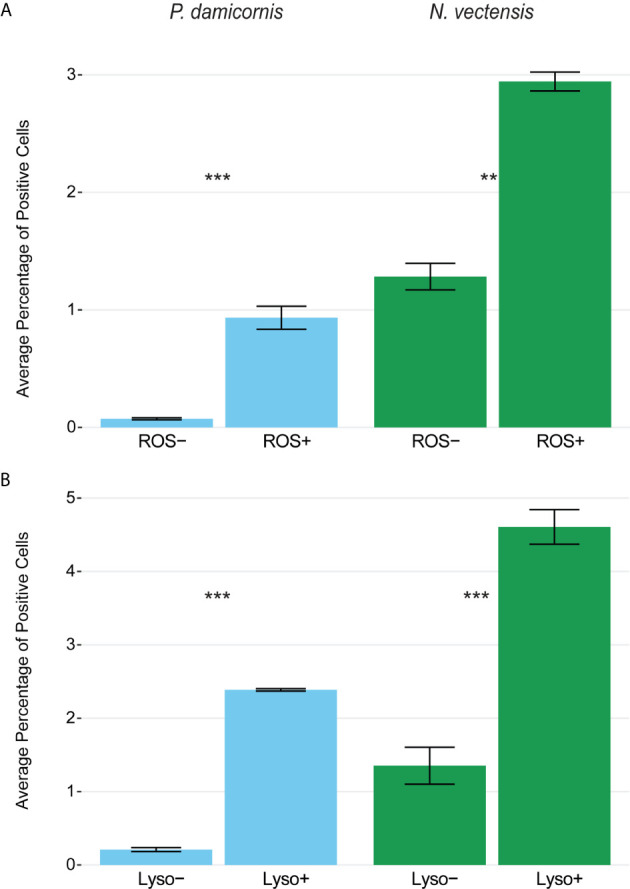
Phagocytic cells are associated with immune cell vesicular markers for ROS (CellROX) and lysosomal vesicles (LysoTracker) in *P. damicornis* and *N. vectensis*. For each species, cells were differentially isolated and sorted based on ROS **(A)** or lysosomal vesicle **(B)** signals, and then co-incubated with beads. **(A)** Comparison of low and high levels of ROS in *P. damicornis* (blue bars) and *N. vectensis* (green bars). There is a significant increase in the number of cells engulfing beads in samples associated with high ROS levels (*P. damicornis* p-value < 0.001; *N. vectensis* p-value < 0.01). **(B)** Comparison of low and high levels of lysolitic vesicle signaling in *P. damicornis* (blue bars) and *N. vectensis* (green bars). There is a significant increase in the number of cells engulfing beads in samples associated with high lysosomal levels (*P. damicornis* p-value < 0.001; *N. vectensis* p-value < 0.001). P-value marks: **p < 0.01, ***p < 0.001.

To test whether the phagocytic cells are enriched with markers associated with mammalian phagocyte markers such as low pH lysosomes and ROS (61, 66, 73), we used markers for the presence of lysosomal vesicles (LysoTracker) and ROS (CellROX), and sorted cells based on high and low amounts of these markers. Rates of bead engulfment are associated with markers for lysosomal vesicles and ROS (**Figures 5**, **S5**). In *N. vectensis*, cells with high lysosome and ROS signal have significantly higher rates of bead engulfment than those with low signal (lysosome signal p-value < 0.001, ROS signal p-value = 0.001; **Figure 5A**). Similar results were obtained with *P. damicornis* cell populations (**Figure 5B**). Collectively, these results suggest that cell sorting based on both high lysosomal vesicle signal and ROS signal can significantly enrich for phagocytic cell populations.

## Discussion

Innate immunity is required to maintain organismal health. Phagocytosis is an essential component of innate immunity and plays a critical role in both the maintenance and re-establishment of homeostasis (74, 75). Now more than ever, we need to have a better understanding of these mechanisms, as the climate change crisis has drastically reduced global coral reef biomass and diversity (10–14). Through the characterization and development of live cell-based assays for phagocytosis, we will be able to both better understand the foundation of coral immune health and establish sensitive rapid diagnostic tools for assessing coral health (6).

In this study, we demonstrate that specific cell populations in two hexacorallians are competent to actively engulf bacteria and carboxylated beads. Upon phagocytosis, we observed low pH in lysosomal vesicles along with increased ROS activity indicative of a respiratory burst associated with lysosome mediated degradation. These markers have previously been used in classical and non-classical model organisms for characterizing phagocytosis. Here we have adapted their use to functionally characterize hexacorallian phagocytosis (61, 72, 76).

### Putative Phagocytes in Hexacorallians

Many invertebrates have specialized types of immune cells that are capable of responding to a diverse range of stressors (77, 78). Historically, hexacorallians have been documented to have at least two distinct types of immune cells, and recently through the use of single cell analysis two putative immune cell populations have been identified in a scleractinian coral (79, 80). Our analyses similarly support the presence of at least two types of putative phagocytes in *P. damicornis* and two types of putative phagocytes in *N. vectensis* (**Figures 1A-E**). In *P. damicornis*, round granular cells, as well as a population of irregularly shaped amoeboid-like cells, phagocytose both beads and bacteria (**Figures 1A, B, E**). In previous studies on wound healing and disease response in *P. damicornis* there was no evidence of phagocytes migrating to the site of a wound and the constituent expression of immune factors was found to be low (19, 81). Additionally, in a transcriptomic study of Pocilloporid corals exposed to lipopolysaccharide (LPS) treatment, differential gene expression was low with only 167 genes differentially expressed post LPS exposure (82). These results suggest that *P. damicornis* has low levels of immune reactivity, and migratory phagocytes may not be involved in eliciting an inflammatory response during wound healing.

In contrast, we observed that both *P. damicornis* and *N. vectensis* phagocytes are able to actively phagocytose beads, bacteria, and fungal antigens with comparable percentages of cells (**Figures 2** **–** **4**), as well as phagocytose self-damaged cells (**Figure 3**). We also observed hydrolysis by proteases of ovalbumin in comparable percentages (**Figures 2C, D**), suggesting that the cells are actively degrading the phagocytic assay particles. However, phagocytosis, and other previously measured components of innate immunity, may not be the only mechanisms that are critical for *P. damicornis* immune function (6, 19, 28, 30, 32, 37, 83). A previous study on *P. damicornis* ectodermal cells found that they express a unique antimicrobial peptide called damicornin (84),. This suggests *P. damicornis* also has taxon-specific mechanisms that are important to cellular immune function.

In *N. vectensis*, nematosomes have previously been shown to engulf particles (62). Structurally, nematosomes are bundles of cnidocyte stinging cells and putative vacuolated phagocytes that are able to migrate through *N. vectensis* tissues. The function of these motile multicellular structures is hypothesized to bridge both digestion and innate immunity (62). In our study, we found that nematosomes are competent to phagocytose both beads and bacterial and fungal antigens, are capable of hydrolysis by proteases to break down foreign pathogens and have increased ROS activity, with a concomitant decrease in lysosomal pH indicative of lysosomal mediated degradation. Thus, our results provide further functional support for the hypothesis that functional nematosomes contain cell types that are likely involved in innate immunity (**Figures 1** **–** **3**, **S2**, **S3**). Further, we also identified a unique amoeboid shaped cell type that is also competent to actively phagocytose beads and antigens for bacteria and fungi, indicating that at least two cell types are involved in this process.

### Phagocytosis Is Distinct From Pinocytosis in Hexacorallians

Previous studies have shown that pinocytosis is ubiquitous in hexacorallian tissues (85–87). Our results support this view. We observed that the majority of cells analyzed were competent for the rapid uptake of FITC-labelled dextran sugars (**Figures 4** and **S4**). In contrast, we observed only 10-30% of the total cell population analyzed was competent for large particle phagocytosis. Phagosomes in these cell populations also showed a concomitant vesicular pH decrease and upregulation of ROS production, indicating the presence of unique phagocyte populations. Phagocytosis and pinocytosis are linked processes that are defined by the passive or active transport of target particle(s) as well as relative particle size (88). While some aspects of phagocytosis and pinocytosis are closely linked, our results show that there are distinct functional differences in hexacorallian cell populations competent to perform active phagocytic engulfment.

### A Link Between Phagocytosis and Coral Bleaching Phenomena?

The Scleractinia holobiome has been called the most diverse symbiotic ecosystem in the world because of the dynamic associations that include numerous bacteria, microeukaryotes, viruses, archaea, and the dinoflagellate Symbiodiniaceae (89). Modifications of phagocytic mechanisms could be one of the means by which these diverse endosymbioses have become so prevalent (53, 56, 57, 59, 90–94). For example, during the establishment of symbioses between Symbiodiniaceae and its coral host, Symbiodiniaceae are phagocytosed, but neither degraded nor exocytosed (92–94). Interestingly, during heat stress, a burst of ROS production from the Symbiodiniaceae intracellular symbiont is a primary mechanism for dysbiosis (55, 95, 96). While there is still discussion whether symbiont ROS production is the initiating factor for bleaching, it is clear that ROS production plays an important role in dysbiosis (97). In multiple species of corals, bleaching has also been shown to activate many important immune pathways such as the melanin and prophenoloxidase cascade, with increased expression of these proteins in phagocytic cells (6, 20, 33, 36, 38). This is further supported by our observations of increased phagocytic activity upon exposure to heat-damaged cells of the same genotype. It is clear that immune response pathways are closely associated with both dysbiosis and coral bleaching phenomena. Future investigation should focus on the interplay of the core phagocytosis and exocytosis mechanisms that underlay the intersection of dysbiosis and coral bleaching processes.

## Conclusions

In this study we showed that hexacorallians have specialized phagocytes that are competent to selectively engulf bacterial and fungal antigens, beads, and self damaged cells. Both *N. vectensis* and *P. damicornis* have cell populations that perform phagocytosis. These phagocytic cell populations also show classic hallmarks of lysosomal mediated degradation evidenced by the decrease in vesicular pH upon bacterial engulfment in association with high ROS production, as well as the presence of protease-driven hydrolysis. Additionally, we show that inhibition of actin cytoskeletal rearrangements significantly diminishes phagocytosis without impeding dextran pinocytosis, showing that target particle engulfment and upregulation of lysosomal mediated degradation is a functional attribute of hexacorallian phagocytes. While the role of coral bleaching mechanisms was not explicitly investigated in this study, our findings show that ROS production occurs in phagocytic cells, and that cells damaged by heat stress are phagocytosed. Our results support further investigation of the relationship between immune cell function, intracellular ROS production, cell damage, and coral bleaching.

## Data Availability Statement

The raw data supporting the conclusions of this article will be made available by the authors, without undue reservation.

## Author Contributions

NT-K, BR, CVP, and GAS conceived and designed the research study. MTC, SE, ST, OG-Y, UH, and WEB assisted with microscopy. GAS, BR, SE, ST, and OG-Y ran the experiments. UH ran the ImageStream analysis. All authors assisted with the writing and editing. All authors contributed to the article and approved the submitted version.

## Funding

NT-K would like to acknowledge the University of Miami Research Awards in Natural Sciences and Engineering for seed funding to begin this research. Additionally, NT-K and BR were supported by NSF-BSF Integrative and Organismal Systems (IOS) Grant: BSF grant number 2019647, NSF grant number 1951826. BR would like to thank Alex and Ann Lauterbach for funding the Comparative and Evolutionary Immunology Laboratory. The work of BR was supported by Israel Science Foundation (ISF) numbers: 1416/19 and 2841/19. BR has received funding from European Research Council (ERC) under the European Union’s Horizon 2020 research and innovation program under grant agreement No. 948476. BR has received funding from a HFSP grant (RGY0085/2019).

## Conflict of Interest

The authors declare that the research was conducted in the absence of any commercial or financial relationships that could be construed as a potential conflict of interest.

## Publisher’s Note

All claims expressed in this article are solely those of the authors and do not necessarily represent those of their affiliated organizations, or those of the publisher, the editors and the reviewers. Any product that may be evaluated in this article, or claim that may be made by its manufacturer, is not guaranteed or endorsed by the publisher.

## Supplementary Material

Supplementary Figure 1 FACS gating strategies for identifying phagocytic cells in *P. damicornis* and *N. vectensis*. All comparisons are of green emission with a green filter (x-axis) to far-red emission (y-axis) that was used to identify host cells using LysoTracker in *P. damicornis* and CellTrace in *N. vectensis*. **(A, B)** Plots of *P. damicornis* show an example gating strategy selecting for fluorescent carboxylated bead-engulfed cells, compared with the untreated sample. **(C, D)** Plots of *N. vectensis* show an example gating strategy selecting for pHrodo™ Green *S. aureus* Bioparticle-engulfed cells, which is enriched compared to the untreated sample.

Supplementary Figure 2 Bacteria*-*positive cells with 3D analysis and ImageStream in *N. vectensis*. Images of isolated cells taken by confocal microscopy. Cells labeled with CellTrace Far Red and positive to pHrodo Green *S. aureus* Bioparticles. **(A)** CellTrace in left panels, pHrodo Green *S*. *aureus* Bioparticles in central panels, and right panels are a combination of both, and confocal microscopy PMT. *S. aureus* presents green fluorescence after the fusion of the bacteria with the lysolitic vesicle, which leads to a decrease in pH. This is not seen in the free bacteria not internalized by the cells and can be seen in gray with PMT, but not with fluorescence (**A**, right panels). Bars represent 5 µm. **(B)** 3D analysis of 40 confocal images done in a Z-stack on the far red and green channels. of the same confocal analysis of the cells in panels A (upper and lower). Again, due to the pHrodo conjugation, only the *S. aureus* bacteria is seen after internalization and fusion to a presumed phagolysosome. The grid represents 10 µm for the upper 3D image and 4 µm for the lower 3D image. **(C)** Inserts of ImageStream analysis of *N. vectensis* cell stained with CellTrace (red) and pHrodo Green *S*. *aureus* Bioparticles. The images are examples of the gated double positive population (as shown in **Supplemental Figure 1D**) for validation of particles engulfed by the red cells. Fluorescent bacteria can be seen in concentrated areas of the cells, suggesting low pH phagolysosomes. Scale bar 7 μm.

Supplementary Figure 3 Validation of beads engulfment with 3D analysis and ImageStream in *N. vectensis*. Images of isolated example cells taken by confocal microscopy. Cells were labeled with CellTrace Far Red and were positive to green, fluorescent beads. **(A)** CellTrace Far Red in left panels, green beads in central panels, and right panels are a combination of both, and confocal microscopy PMT. Bars represent 5 µm. **(B)** 3D analysis of 40 confocal images done in a Z-stack on the far red and green channels of the same confocal analysis of the cells in panel A. Two views, from the side (left panel) and from above (right panel), showing that the bead is internalized in the cell. Grid represents 5 µm. **(C)** Inserts of ImageStream analysis of *N. vectensis* cells stained with CellTrace (red) and green fluorescent beads. The images are examples of the gated double positive population (as shown in **Supplementary Figure 1B**) for validation of beads intake by the red cells. Scale bar 7 μm.

Supplementary Figure 4 Cells engaging in phagocytosis are exclusive from those that engage in large molecule pinocytosis. **(A)** Percentages of *P. damicornis* and *N. vectensis* cells engulfing beads of two different sizes; the bead sizes of 1 and 4 µm in diameter were used here. While similar percentages of cells were found to engulf beads of both sizes compared to the population engaging in pinocytosis of dextran molecules, a reduction in bead engulfment was seen in both species in the 4 µm bead size. **(B)** Percentages of *P. damicornis* and *N. vectensis* cells consuming dextran molecules of four different molecular weights. **(C)** FACS analysis of *N. vectensis* cells exposed to FITC-conjugated dextran and red fluorescent beads simultaneously. The large population residing within the bottom right quadrant are those that have consumed dextran and is completely separated from the linear population of cells spreading across the upper region of the y-axis that consists of cells with engulfed red beads. This suggests no correlation between phagocytosis and pinocytosis. **(D)** Minor reductions in bead engulfment are observed in both species when cells are exposed to trypan blue, which is known to quench external fluorescence, meaning the remaining percentage of engulfed beads are internalized within the cell.

Supplementary Figure 5 FACS-isolated phagocytic cells associated with immune cell vesicular markers. For each species, cells with high and low ROS or lysosome staining were sorted and co-incubated with beads at a ratio of 1:4 cells: beads for analysis of phagocytosis (as done in **Figure 5**). **(A)** Examples of the analysis and gating of the 4 groups co-incubated with beads in *P. damicornis.* **(B)** Examples of the analysis and gating of the 4 groups co-incubated with beads in *N. vectensis.* In both animals, phagocytosis is higher in CellROX or LysoTracker positive sorted cells (or high stain signal) compared to those with low expression (low stain signal). This suggests that the phagocytic cells enriched for lysolitic vesicles and ROS are comparable to mammalian phagocytic cells.

Supplementary Video 1 Video of *P. damicornis* phagocytic cell with engulfed beads. Top center area of the field of view shows a cell with two fluorescent, green beads internalized. The amoeboid-shaped cell is approximately 5 and 10 µm in diameter, and scarcely shows pseudopodial movement on the upper and lower corners of the cell. In contrast to the internalized cells, there are non-engulfed beads that are flowing through the media and move rapidly across the field of view. There are also large (~10 µm in diameter) Symbiodiniaceae cells that are highly autofluorescent due to green fluorescent proteins. The phagocytic cell shown here shows the internalization of green beads and suggests that these cells can simultaneously phagocytose multiple non-self-particles.
