# Supplementary figures and images for: Functional Characterization of Hexacorallia Phagocytic Cells
Source: Front Immunol. 2021 Jul 26;12:662803. doi: 10.3389/fimmu.2021.662803 (PMC8350327; doi:10.3389/fimmu.2021.662803)

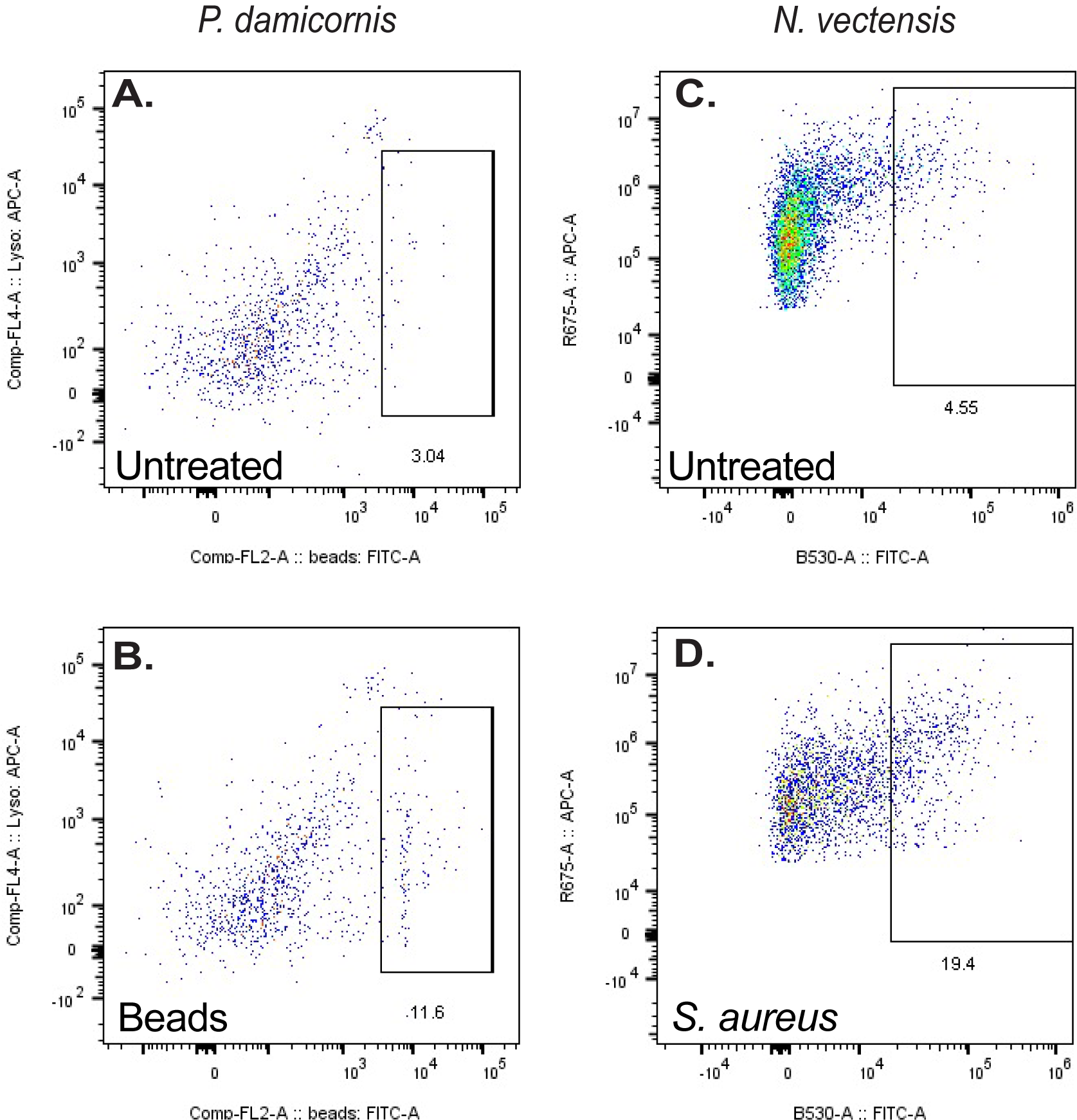

Supplement: Supplementary Figure 1 — FACS gating strategies for identifying phagocytic cells in P. damicornis and N. vectensis. All comparisons are of green emission with a green filter (x-axis) to far-red emission (y-axis) that was used to identify host cells using LysoTracker in P. damicornis and CellTrace in N. vectensis. (A, B) Plots of P. damicornis show an example gating strategy selecting for fluorescent carboxylated bead-engulfed cells, compared with the untreated sample. (C, D) Plots of N. vectensis show an example gating strategy selecting for pHrodo™ Green S. aureus Bioparticle-engulfed cells, which is enriched compared to the untreated sample. [file Image_1.tif]

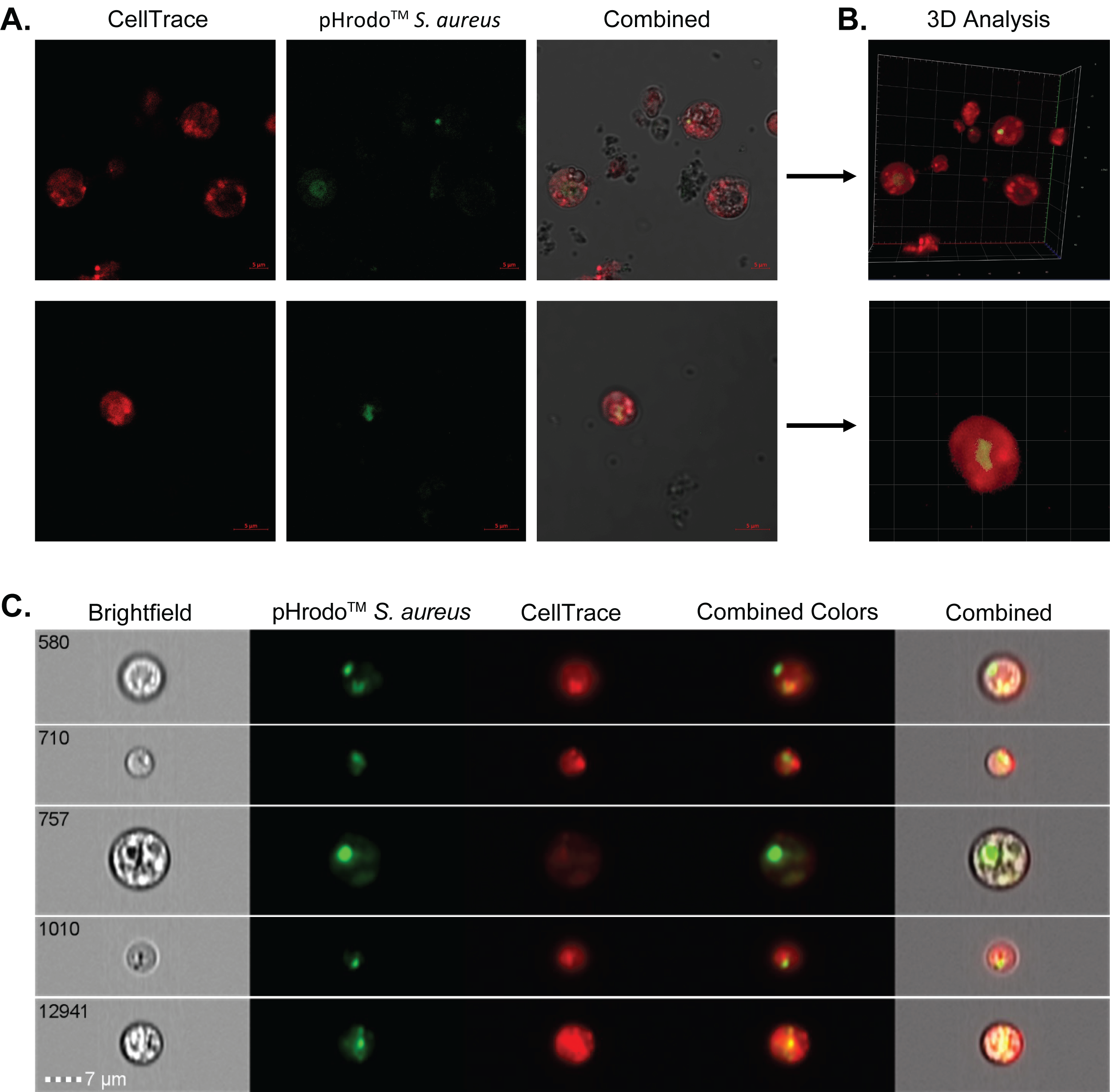

Supplement: Supplementary Figure 2 — Bacteria-positive cells with 3D analysis and ImageStream in N. vectensis. Images of isolated cells taken by confocal microscopy. Cells labeled with CellTrace Far Red and positive to pHrodo Green S. aureus Bioparticles. (A) CellTrace in left panels, pHrodo Green S. aureus Bioparticles in central panels, and right panels are a combination of both, and confocal microscopy PMT. S. aureus presents green fluorescence after the fusion of the bacteria with the lysolitic vesicle, which leads to a decrease in pH. This is not seen in the free bacteria not internalized by the cells and can be seen in gray with PMT, but not with fluorescence (A, right panels). Bars represent 5 µm. (B) 3D analysis of 40 confocal images done in a Z-stack on the far red and green channels. of the same confocal analysis of the cells in panels A (upper and lower). Again, due to the pHrodo conjugation, only the S. aureus bacteria is seen after internalization and fusion to a presumed phagolysosome. The grid represents 10 µm for the upper 3D image and 4 µm for the lower 3D image. (C) Inserts of ImageStream analysis of N. vectensis cell stained with CellTrace (red) and pHrodo Green S. aureus Bioparticles. The images are examples of the gated double positive population (as shown in Supplemental Figure 1D ) for validation of particles engulfed by the red cells. Fluorescent bacteria can be seen in concentrated areas of the cells, suggesting low pH phagolysosomes. Scale bar 7 μm. [file Image_2.tif]

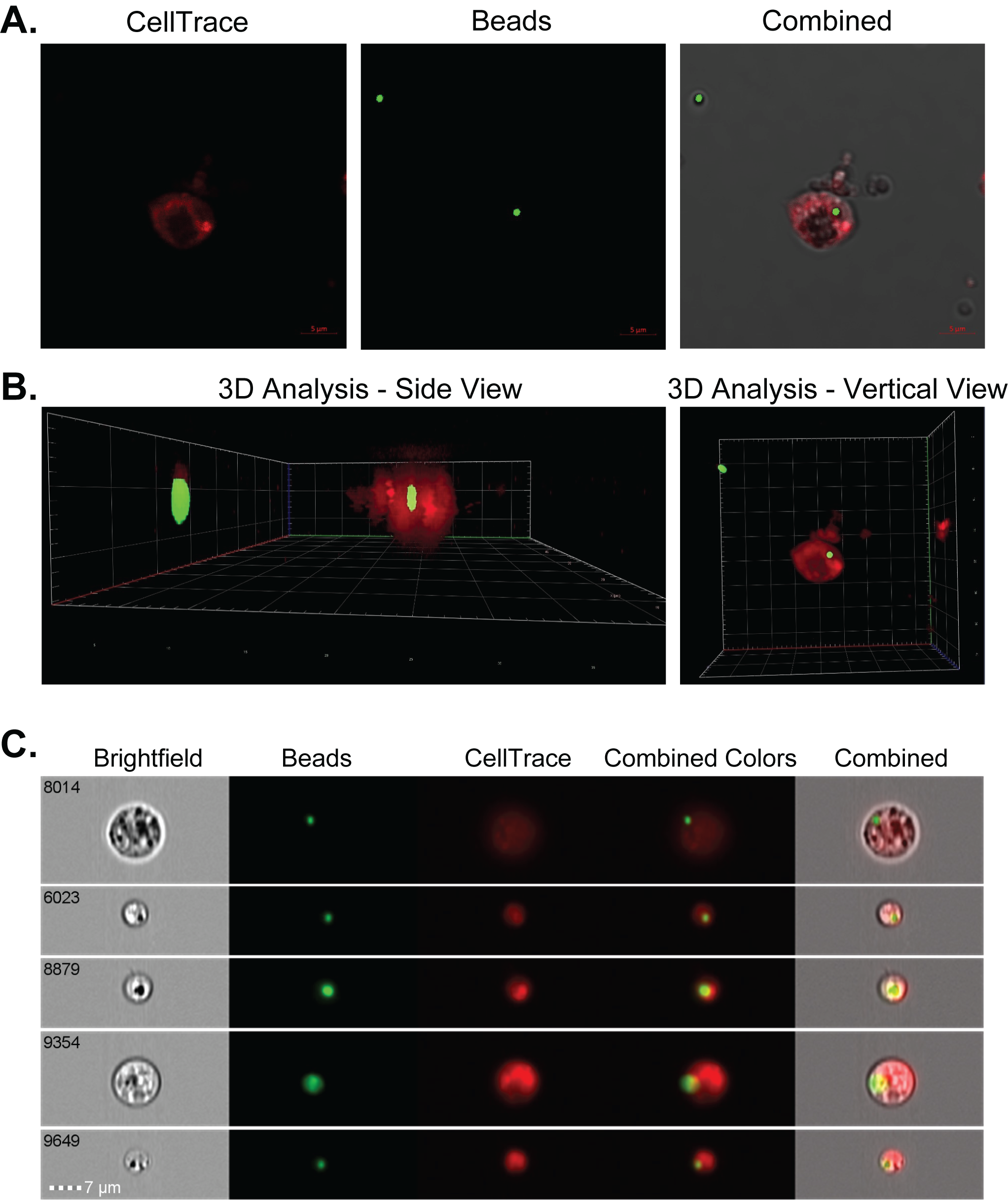

Supplement: Supplementary Figure 3 — Validation of beads engulfment with 3D analysis and ImageStream in N. vectensis. Images of isolated example cells taken by confocal microscopy. Cells were labeled with CellTrace Far Red and were positive to green, fluorescent beads. (A) CellTrace Far Red in left panels, green beads in central panels, and right panels are a combination of both, and confocal microscopy PMT. Bars represent 5 µm. (B) 3D analysis of 40 confocal images done in a Z-stack on the far red and green channels of the same confocal analysis of the cells in panel A. Two views, from the side (left panel) and from above (right panel), showing that the bead is internalized in the cell. Grid represents 5 µm. (C) Inserts of ImageStream analysis of N. vectensis cells stained with CellTrace (red) and green fluorescent beads. The images are examples of the gated double positive population (as shown in Supplementary Figure 1B ) for validation of beads intake by the red cells. Scale bar 7 μm. [file Image_3.tif]

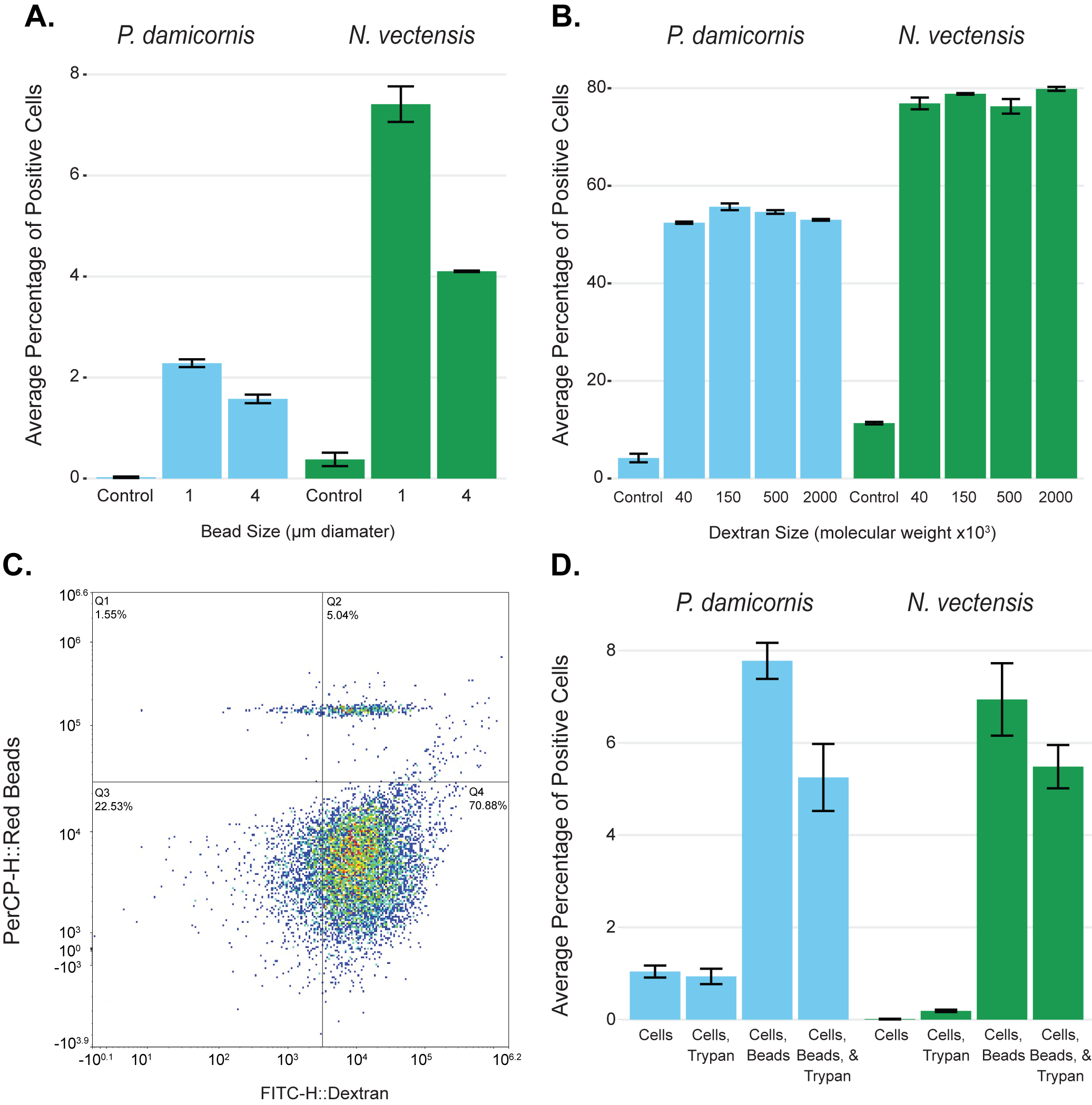

Supplement: Supplementary Figure 4 — Cells engaging in phagocytosis are exclusive from those that engage in large molecule pinocytosis. (A) Percentages of P. damicornis and N. vectensis cells engulfing beads of two different sizes; the bead sizes of 1 and 4 µm in diameter were used here. While similar percentages of cells were found to engulf beads of both sizes compared to the population engaging in pinocytosis of dextran molecules, a reduction in bead engulfment was seen in both species in the 4 µm bead size. (B) Percentages of P. damicornis and N. vectensis cells consuming dextran molecules of four different molecular weights. (C) FACS analysis of N. vectensis cells exposed to FITC-conjugated dextran and red fluorescent beads simultaneously. The large population residing within the bottom right quadrant are those that have consumed dextran and is completely separated from the linear population of cells spreading across the upper region of the y-axis that consists of cells with engulfed red beads. This suggests no correlation between phagocytosis and pinocytosis. (D) Minor reductions in bead engulfment are observed in both species when cells are exposed to trypan blue, which is known to quench external fluorescence, meaning the remaining percentage of engulfed beads are internalized within the cell. [file Image_4.tif]

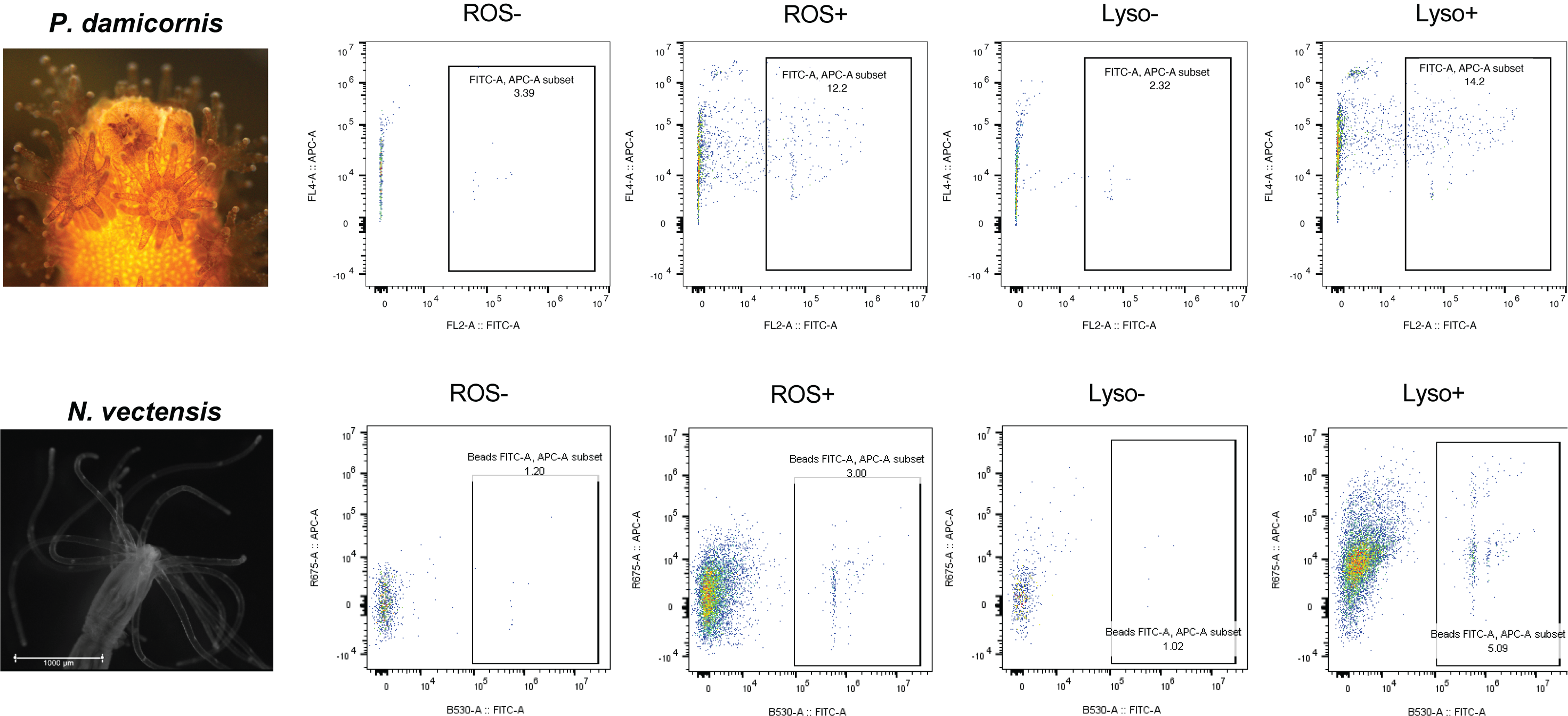

Supplement: Supplementary Figure 5 — FACS-isolated phagocytic cells associated with immune cell vesicular markers. For each species, cells with high and low ROS or lysosome staining were sorted and co-incubated with beads at a ratio of 1:4 cells: beads for analysis of phagocytosis (as done in Figure 5 ). (A) Examples of the analysis and gating of the 4 groups co-incubated with beads in P. damicornis. (B) Examples of the analysis and gating of the 4 groups co-incubated with beads in N. vectensis. In both animals, phagocytosis is higher in CellROX or LysoTracker positive sorted cells (or high stain signal) compared to those with low expression (low stain signal). This suggests that the phagocytic cells enriched for lysolitic vesicles and ROS are comparable to mammalian phagocytic cells. [file Image_5.tif]
